# Authors' Response to Editorial: Maternal Death Surveillance and Response: A Tall Order for Effectiveness in Resource-Poor Settings

**DOI:** 10.9745/GHSP-D-17-00407

**Published:** 2017-12-28

**Authors:** Helen Smith, Charles Ameh, Pamela Godia, Judith Maua, Kigen Bartilol, Patrick Amoth, Matthews Mathai, Nynke van den Broek

**Affiliations:** a Centre for Maternal and Newborn Health, Liverpool School of Tropical Medicine, Liverpool, UK.; b Liverpool School of Tropical Medicine Kenya Office, Nairobi, Kenya.; c Kenya National Maternal and Perinatal Death Surveillance and Response Secretariat, Nairobi, Kenya.; d Reproductive Maternal Health Services Unit, Ministry of Health, Nairobi, Kenya.; e Division of Family Health, Ministry of Health, Nairobi, Kenya.

See related articles by Koblinsky and by Smith et al.

We thank Marge Koblinksy for her considered editorial^1^ on the Maternal Death Surveillance and Response (MDSR) approach used in Kenya and lessons learned, described in our recent article published in GHSP.^2^ Her views on the potential effectiveness of MDSR in resource-limited settings, however, seem pessimistic.

Firstly, Koblinsky argues that MDSR is too complicated and demanding in low- and middle-income countries, and should be abandoned in favour of investment in lifesaving interventions. We argue, however, that investment must be made in ensuring availability of care as well as quality of this care for interventions to be lifesaving. Most maternal deaths in low- and middle-income countries result from obstetric complications. The care packages to prevent and manage these complications are established and evidence-based. Most maternal deaths occur because complications are not recognized on time, women do not receive these interventions on time, or care given may be substandard.

Secondly, Koblinsky criticizes the assessment of factors contributing to maternal deaths in the national report from Kenya—incorrect management, insufficient monitoring, and delay in taking action when needed—as being too general. Yet these are exactly the reasons why women die. For example, the failure to identify severe hemorrhage early and to take timely and adequate action is what leads to death in women with hemorrhage in many cases. It is only by understanding these factors through a systematic review process such as maternal death audit that health care providers can identify what actions need to be undertaken to improve the quality of care and outcomes. Similarly, by aggregating information across settings, regional and national governments can identify cross-cutting themes and formulate priority recommendations, such as the need to strengthen blood transfusion services. In places where MDSR is currently implemented including the Republic of South Africa, Malaysia, and several states in India, there is emerging evidence that this results in measurable improvements in availability and quality of care with renewed priority setting and investment in maternal and newborn health.^3^^,^^4^

The editorial also levels criticism more generally at the country-level efforts in Kenya and remarks that the results presented fall short of what is needed. In the first year of MDSR implementation at the national level using the Confidential Enquiry into Maternal Deaths (CEMD) approach, the decision was to start with the identification and review of maternal deaths in major comprehensive emergency obstetric care facilities. A total of 52% of all maternal deaths reported to have occurred in these facilities were included in the report. In a country with 62% of births occurring in a health care facility, the review process discovered that maternal deaths occurring at the health facility level were underreported and that the District Health Information System 2 (DHIS 2) database did not capture deaths occurring at the community level. In response, the government is reorganizing the maternal death surveillance system to ensure that *all* maternal deaths are reported through DHIS 2.^5^ Furthermore, criticism of the lack of action following the recommendations made in the report is premature—the report has only just been completed, and the Ministry of Health (MOH) will formally launch it by the end of 2017. We can confirm that for the first time in Kenya, specific recommendations for action by various stakeholders (e.g., the MOH, county governments, professional associations, and civil society) with indicators and targets to guide the ‘Response’ have been produced. This is a significant step forward

Koblinsky summarizes the history of maternal death audit and its evolution into CEMD in high-income countries. However, her point that MDSR is likely to be more successful in countries with low maternal mortality ratios (MMRs) is not based on sound evidence. In the United Kingdom, the use of maternal death reviews started small as a practitioner-led activity in Rochdale, in northwest England, between 1931 and 1934. Prior to this, the MMR in Rochdale was estimated to be 900 per 100,000 live births, twice the national average. In 1934, at a time of severe economic depression, because of actions taken following the Rochdale Enquiry, the MMR decreased to 280, “without any alteration in personnel or any substantial increase in public expenditure.”^6^ The first full national enquiry into maternal deaths in the United Kingdom was not conducted until 30 years later in 1952 and has successfully continued to date as an important quality improvement process.

The impetus and commitment that drove the process in Kenya was from the highest administrative level, the Cabinet Secretary for Health, and supported by the professional medical and midwifery associations and regulatory bodies. The running of the committee was not a “gray area.” There was no precedent, so it was initially challenging to get the committee up and running. Despite these challenges, the committee is well-established and has terms of reference agreed by all stakeholders and approved by the MOH. The committee is actively led and managed by the national MDSR secretariat situated within the MOH.

In our article, we document implementation of MDSR in a ‘real-life’ setting, reporting on experiences and lessons learned working in partnership with the MOH in Kenya to implement their previously agreed guidelines on Maternal and Perinatal Death Surveillance and Response (MPDSR) based on the World Health Organization MDSR guidelines.^7^ We do not advocate that MDSR is the *only* solution to reducing maternal mortality, nor do we suggest it is easy to implement. We do, however, provide a careful analysis of what needs to be in place for successful implementation and what could facilitate the maturation of the process in a country like Kenya. We certainly acknowledge that there is still more to do. Aware that otherlow- and middle-income countries are currently embarking on establishing MDSR, we wish to share the important lessons learned in Kenya.
